# New Insight regarding *Legionella* Non-*Pneumophila* Species Identification: Comparison between the Traditional *mip* Gene Classification Scheme and a Newly Proposed Scheme Targeting the *rpoB* Gene

**DOI:** 10.1128/Spectrum.01161-21

**Published:** 2021-12-15

**Authors:** Maria Rosaria Pascale, Silvano Salaris, Marta Mazzotta, Luna Girolamini, Giulia Fregni Serpini, Laura Manni, Antonella Grottola, Sandra Cristino

**Affiliations:** a Department of Biological, Geological, and Environmental Sciences, University of Bologna, Bologna, Italy; b Regional Reference Laboratory for Clinical Diagnosis of Legionellosis, Molecular Microbiology and Virology Unit, University Hospital-Policlinico Modena, Modena, Italy; University at Albany, State University of New York

**Keywords:** *Legionella* non-*pneumophila* species, *Legionella* identification, *mip* and *rpoB* gene sequencing, nucleotide identity percentage, interspecies and intraspecies variation interval, classification scheme, *Legionella* spp., whole-genome sequencing, environmental microbiology, gene classification scheme, genotyping, surveillance

## Abstract

The identification of *Legionella* non-*pneumophila* species (non-*Lp*) in clinical and environmental samples is based on the *mip* gene, although several studies suggest its limitations and the need to expand the classification scheme to include other genes. In this study, the development of a new classification scheme targeting the *rpoB* gene is proposed to obtain a more reliable identification of 135 *Legionella* environmental isolates. All isolates were sequenced for the *mip* and *rpoB* genes, and the results were compared to study the discriminatory power of the proposed *rpoB* scheme. Complete concordance between the *mip* and *rpoB* results based on genomic percent identity was found for 121/135 (89.6%) isolates; in contrast, discordance was found for 14/135 (10.4%) isolates. Additionally, due to the lack of reference values for the *rpoB* gene, inter- and intraspecies variation intervals were calculated based on a pairwise identity matrix that was built using the entire *rpoB* gene (∼4,107 bp) and a partial region (329 bp) to better evaluate the genomic identity obtained. The interspecies variation interval found here (4.9% to 26.7%) was then proposed as a useful sequence-based classification scheme for the identification of unknown non-*Lp* isolates. The results suggest that using both the *mip* and *rpoB* genes makes it possible to correctly discriminate between several species, allowing possible new species to be identified, as confirmed by preliminary whole-genome sequencing analyses performed on our isolates. Therefore, starting from a valid and reliable identification approach, the simultaneous use of *mip* and *rpoB* associated with other genes, as it occurs with the sequence-based typing (SBT) scheme developed for Legionella pneumophila, could support the development of multilocus sequence typing to improve the knowledge and discovery of *Legionella* species subtypes.

**IMPORTANCE** *Legionella* spp. are a widely spread bacteria that cause a fatal form of pneumonia. While traditional laboratory techniques have provided valuable systems for Legionella pneumophila identification, the amplification of the *mip* gene has been recognized as the only useful tool for *Legionella* non-*pneumophila* species identification both in clinical and environmental samples. Several studies focused on the *mip* gene classification scheme showed its limitations and the need to improve the classification scheme, including other genes. Our study provides significant advantages on *Legionella* identification, providing a reproducible new *rpoB* gene classification scheme that seems to be more accurate than *mip* gene sequencing, bringing out greater genetic variation on *Legionella* species. In addition, the combined use of both the *mip* and *rpoB* genes allowed us to identify presumed new *Legionella* species, improving epidemiological investigations and acquiring new understanding on *Legionella* fields.

## INTRODUCTION

*Legionella* spp. have been described as the causative agent of legionellosis in humans. The term refers to two main form of diseases, Legionnaires’ disease, a severe multisystem disease involving pneumonia, and Pontiac fever, a nonpneumonic form, acute, self-limiting influenza-like illness. Additionally, extrapulmonary (e.g., sinusitis, hip wound infection, and prosthetic valve endocarditis) and asymptomatic forms are reported (1–3). The *Legionella* genus includes Gram-negative aerobic bacteria widely found in both natural and artificial aquatic environments, where they can multiply inside free-living amoebae, protozoa, and biofilms, exploiting them as a source of nourishment and protection (4–6).

Currently, 66 *Legionella* species have been identified to date, and about half of them are linked with human infection, and some species contain more than one serogroup (7, 8). The most studied species is Legionella pneumophila (*Lp*), which comprises 16 serogroups; the majority of cases, clusters, and outbreaks are attributable to serogroup 1 (Sg1). Other *Legionella* non*-pneumophila* species (non-*Lp*) are less studied and less commonly associated with human disease; thus, they remain undiagnosed due to limits of current diagnostic methods, which are more specific and sensitive for *Lp.* Indeed, the commonly used diagnostic method is the detection of a urinary antigen that is more sensitive for *Lp* Sg1 and does not permit the detection of *Lp* non-Sg1 or other *Legionella* species (9).

Among non-*Lp* species, Legionella longbeachae is the leading cause of infection in Australia and New Zealand, and potting soil mixes are considered the main source of infection (8, 10). Legionella anisa, in addition to being isolated with *Lp*, is associated with several cases of legionellosis and coinfection (11–16), while Legionella rubrilucens was isolated from pneumonia patients coinfected with *Lp* (17).

Considering the broad distribution of *Legionella* and the high incidence of disease, environmental *Legionella* surveillance is an important activity for preventing legionellosis (18). Monitoring of several water sources (water distribution systems, cooling towers, fountains, spas, etc.) remains the main approach to prevent infection and to perform a fast identification of clusters and outbreaks that occur in community, hospital, and travel settings. Therefore, the possibility of rapidly identifying *Legionella* spp. with highly specific and sensitive methods represents one of the most important objectives for the control and prevention of *Legionella.*

Over time, numerous methods have been developed for the detection, identification, and typing of *Legionella* spp. both in clinical and environmental samples. The culture of clinical and environmental samples is the gold standard for *Legionella* detection, and the subcultivation of isolated colonies on buffered charcoal yeast extract (BCYE) without l-cysteine (l-cys) is the first step to discriminate *Legionella* from other bacteria. Serological methods, such as the agglutination test, the direct fluorescent antibody (DFA) test, and indirect immunofluorescence assay (IFA), are mostly used for discrimination between non-*Lp* species and *Lp* serogroups (19, 20). Although these methods are commonly used, each of them shows different sensitivity and specificity and various error ranges; the culture technique is time consuming, technically difficult, and requires a long incubation time. However, serological methods lead to the occurrence of false-negative results and cross-reaction between species, limiting their specificity (1, 21).

To overcome these limitations, more rapid and precise identification of *Legionella* spp. can be provided by sequence analyses, which, as simple tools, can reduce the time needed for *Legionella* isolate identification with improved sensitivity and specificity (22, 23). Currently, the gold standard for *Legionella* spp. typing is based on the approaches developed by the European Working Group for *Legionella* Infection (EWGLI) that are represented by a sequence-based typing (SBT) approach for clinical and environmental *Lp* strains (24, 25) and a database based on macrophage infectivity potentiator (*mip*) gene sequencing for non-*Lp* isolates (26, 27). Currently, while for clinical and environmental *Lp* strains, a multilocus typing scheme has been developed by the EWGLI, represented by a SBT approach (24, 25), regarding the non-*Lp* isolates, identification is still based only on *mip* gene sequencing (26, 27), and no recognized and standardized typing approach was developed. Regarding the identification of *Legionella* species, several genetic markers have been proposed, including 16S rRNA, which was subsequently replaced by the *mip* gene, as this gene can overcome the limitations of intraspecies heterogenicity in the 16S rRNA gene (28). However, some species and some environmental isolates could not be confidently discriminated by the *mip* scheme, such as *L. geestiana* or European wild strain LC4381 (29).

Another gene that is widely used for bacterial identification is the *rpoB* gene. This gene encodes a subunit of DNA-dependent RNA polymerase, and mutations in its sequence are known to cause rifampicin resistance. *rpoB* DNAs comprise a highly conserved region throughout bacteria that may be used for bacterial classification (30). It can identify enteric bacteria, Mycobacterium, spirochetes, and *Legionella* species, including some causative agents of Legionnaires’ disease (30, 31). Regarding the identification of non-*Lp*, the nucleotide variation of *rpoB* is able to differentiate these species better than 16S rRNA and *mip* in some cases (31). The partial *rpoB* sequence (300 bp) can guarantee the genotypic classification of *Lp* and blue-white autofluorescent species (31). This region can distinctly differentiate species that share high similarities in their 16S rRNA gene sequences and that cannot be analyzed successfully by *mip* (26, 31). Thus, *rpoB* analysis could clearly differentiate among *Legionella* spp.

Although *rpoB* has higher intraspecies variability, it is widely used for bacterial identification, and it is considered, in some cases, to be the best approach, such as for nontuberculous mycobacteria (NTM) and Acinetobacter. This marker is not sufficient for *Legionella* classification, especially for non-*Lp*, although different studies have already suggested combining *rpoB* with the *mip* gene to identify these species more accurately (32–34). In addition, in the scientific literature, there are reference values for *mip* gene analysis that can be used to determine the inter- and intraspecies nucleotide variation; however, for *rpoB*, there are no works that establish reference intervals (26), and this limits the application of the *rpoB* gene as a marker in the classification scheme.

In the present study, 135 *Legionella* spp. strains recovered from environmental communities were analyzed for *rpoB* gene sequencing, and the results obtained were compared with a *mip* gene sequencing identification scheme to study the discriminatory power of *rpoB* sequences and establish an inter- and intraspecies variation interval to improve the use of the *rpoB* gene as a target for non-*Lp* identification.

## RESULTS

All 135 isolates showed positive growth on BCYE cys^+^ and negative growth on BCYE cys^−^ and tryptone soy agar (TSA) with 5% sheep blood agar. Moreover, the agglutination for *Legionella* species antisera test displayed positive results for 34/135 isolates (25.2%) and ambiguous results for 10/135 (7.4%) isolates; in contrast, most isolates (91/135 [67.4%]) showed negative results for the agglutination test. All of them were then submitted for gene amplification as previously described.

### *mip* and *rpoB* sequencing results.

All isolates (135/135 [100%]) were identified by *mip* and *rpoB* sequencing analysis at the species level as follows: L. anisa 51/135 (37.7%), *L. rubrilucens* 26/135 (19.2%), *L. taurinensis* 22/135 (16.3%), and *L. nautarum* 15/135 (11.1%). The remaining isolates were represented by *L. feeleii* 7/135 (5.2%), *L*. *londiniensis* 7/135 (5.2%), *L*. *quateirensis* 4/135 (3.0%), *L*. *quinlivanii* 1/135 (0.7%), and *L. steelei* 1/135 (0.7%). The positive control was confirmed to belong to L. pneumophila.

Regarding the percentage of genomic identity, complete concordance between *mip* and *rpoB* results was found in 121/135 (89.6%) isolates; in contrast, discordance was returned for 14/135 (10.4%) isolates. In particular, it is possible to evaluate the number of isolates displaying concordance between the *mip* and *rpoB* results, including 49/135 belonging to L. anisa, 26/135 belonging to *L. rubrilucens*, 15/135 belonging to *L. nautarum*, 7/135 belonging to *L. londiniensis*, 1/135 belonging to *L. steelei*, 22/135 belonging to *L. taurinensis*, and 1/135 belonging to *Lp*.

The results obtained by *mip* and *rpoB* gene sequencing and their ranges of matches compared to the reference strains are shown in Table 1, where the 14 isolates with a discrepancy in the nucleotide identity percentage for *mip*, *rpoB*, or both genes are highlighted in bold. Regarding *mip* gene identification, compared with the respective reference strains, our isolates showed a nucleotide identity interval of 98.2% to 100%, with the exception of two L. anisa isolates and one *L. quinlivanii* isolate with nucleotide identities of 96.7% and 96.2%, respectively. However, the *rpoB* gene results showed a nucleotide identity interval of 95.1% to 100%, except for two isolates of L. anisa and four isolates of *L. quateirensis*, which were identical to each other with nucleotide identity percentages of 92.4% and 94.5%, respectively. Moreover, for the seven isolates identified by the *mip* gene as *L. feeleii* (98.2%), a discrepancy with the *rpoB* gene identity results was found, showing a percentage of identity of 95.4% for six isolates and 95.1% for one isolate.

**TABLE 1:** Comparison of *mip* and *rpoB* gene sequence results for match percentage (%), number of mismatches (mm), and interspecies identity and variation percentage with the respective reference strains

		*mip* gene nucleotide identity (%), no. of mm, and nucleotide variation (%)		*rpoB* gene nucleotide identity (%), no. of mm, and nucleotide variation (%)	
		Interspecies identity interval^*a*^	Interspecies variation interval^*a*^	Interspecies identity interval^*b*^	Interspecies variation interval^*b*^
No. of isolates	Reference strain	69.5–96.4%	3.6–30.5%	73.3–95.1%	4.9–26.7%
*n* = 51 L. anisa	ATCC 35292	*n* = 48 100.0%; 0 mm; 0%		*n* = 48 100.0%; 0 mm; 0%	
		***n* = 2** **96.7%; 20 mm; 3.3%**		***n* = 2** **92.4%; 25 mm; 7.6%**	
		*n* = 1 99.8%; 1 mm; 0.2%		*n* = 1 99.4%; 2 mm; 0.6%	
*n* = 26 *L. rubrilucens*	ATCC 35304	100.0%; 0 mm; 0%		100.0%; 0 mm; 0%	
*n* = 22 *L. taurinensis*	NCTC 13314	100.0%; 0 mm; 0%		*n* = 21 100.0%; 0 mm; 0%	
				*n* = 1 97.3%; 9 mm; 2.7%	
*n* = 15 *L. nautarum*	ATCC 49506	100.0%; 0 mm; 0%		100.0%; 0 mm; 0%	
*n* = 7 *L. feeleii*	ATCC 35072	**98.2%; 11 mm; 1.8%**		***n* = 6** **95.4%; 15 mm; 4.6%**	
				***n* = 1** **95.1%; 16 mm; 4.9%**	
*n* = 7 *L. londiniensis*	ATCC 49505	100.0%; 0 mm; 0%		100.0%; 0 mm; 0%	
*n* = 4 *L. quateirensis*	ATCC 49507	**98.2%; 11 mm; 1.8%**		**94.5%; 18 mm; 5.5%**	
*n* = 1 *L. quinlivanii*	ATCC 43830	**96.2%; 23 mm; 3.8%**		**95.7%; 14 mm; 4.3%**	
*n* = 1 *L. steelei*	ATCC BAA2169	99.8%; 1 mm; 0.2%		100.0%; 0 mm; 0%	
Positive control *n* = 1 L. pneumophila	ATCC 33152	99.0%; 6 mm; 1.0%		98.8%; 4 mm; 1.2%	

a Reference 26.

b Based on the 329-bp matrix of the type strain (Fig. 2a and b).

To obtain a reliable identification scheme for the *rpoB* gene in our isolates, it was important to determine the specific intra- and interspecies variation intervals, as has been done for the *mip* sequence-based classification scheme created by Ratcliff et al. (26). Therefore, our attention was focused mainly on the 14 isolates previously described as having higher discrepancies in nucleotide identity percentage.

**FIG 1:**
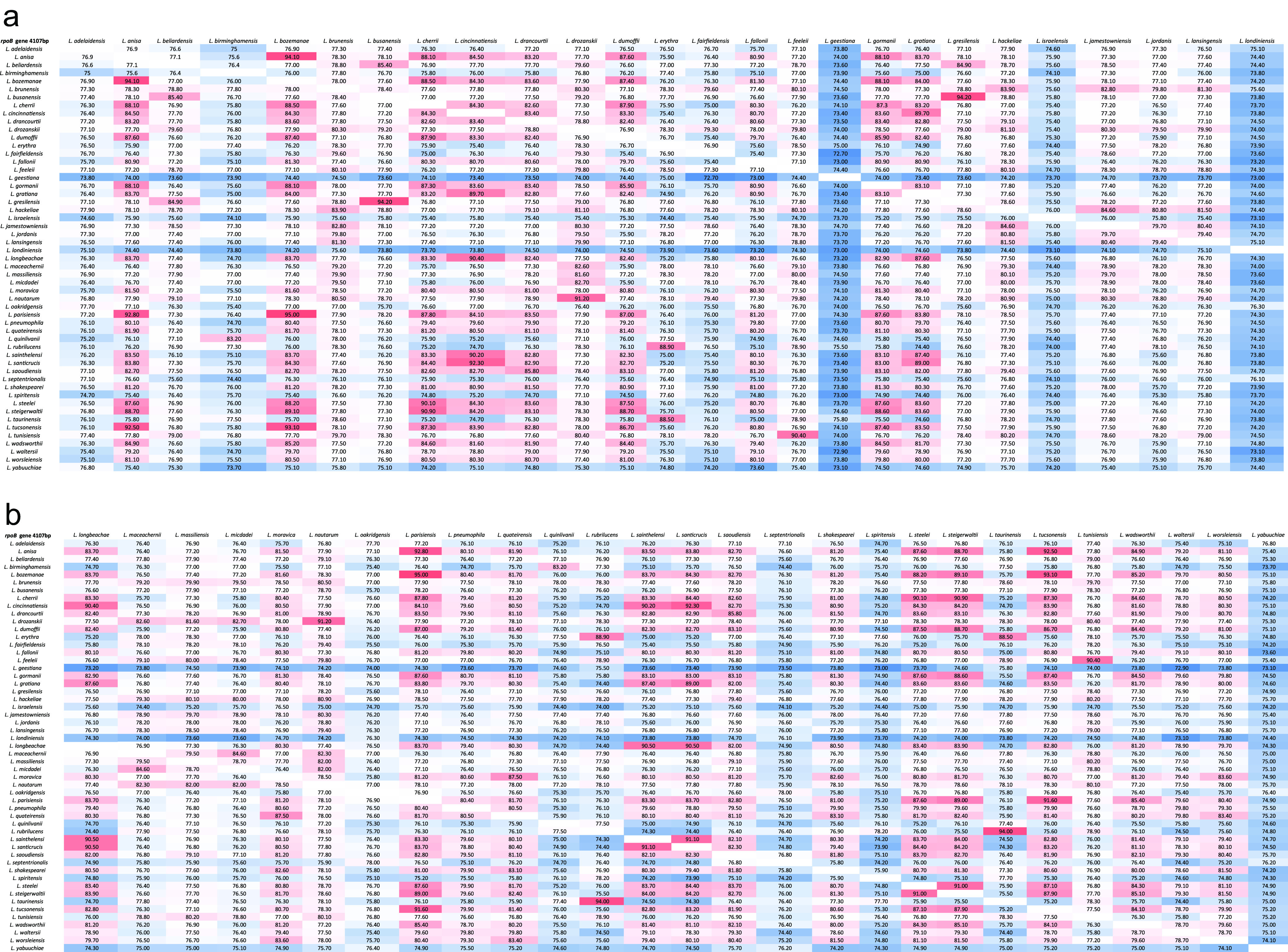
(a, b) Pairwise matrix developed using the entire *rpoB* gene size (4,101 to 4,143 bp) of 53 *Legionella* type strains used to determine the ranges of intra- and interspecies intervals of variation. The heatmap colours represent the range of similarity: from dark red (highest value) to dark blue (lowest value).

A pairwise identity matrix for the entire length of the *rpoB* gene based on 53 reference strains downloaded from NCBI, with a gene size from 4,101 to 4,143 bp, was built (Fig. 1a and b). The matrix returned an interspecies pairwise identity interval of 72.7% to 95.0%. Therefore, the obtained interspecies variation interval was between 5.0% and 27.3%. The calculated intraspecies identity interval was 95.1% to 100%, resulting in an intraspecies variation interval between 0% and 4.9%, which permits the classification of unknown isolates as belonging to the same species.

**FIG 2:**
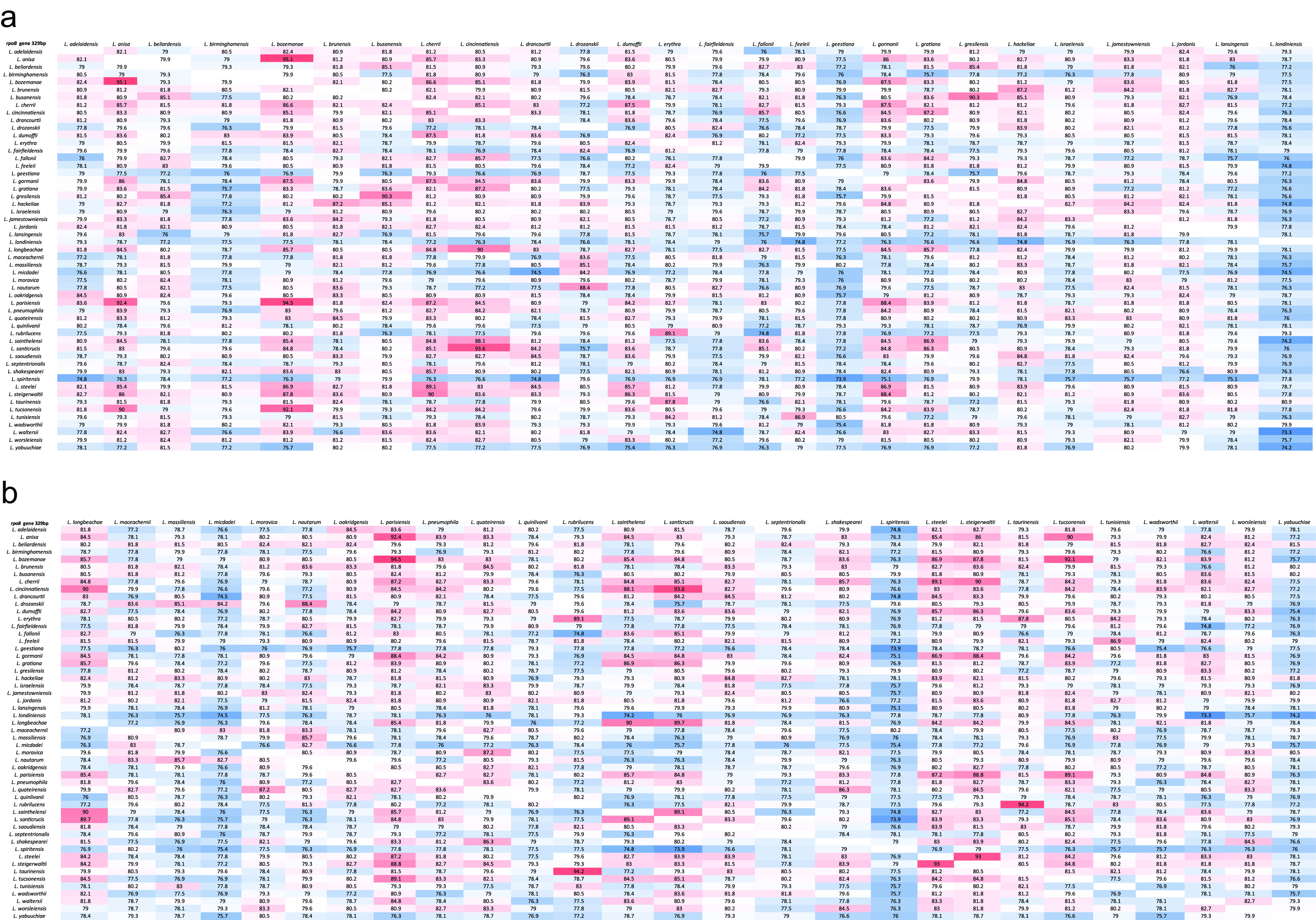
(a, b) Pairwise matrix developed using the selected region of the *rpoB* gene (329 bp) of 53 *Legionella* type strains used to determine the ranges of intra- and interspecies intervals of variation. The heatmap colours represent the range of similarity: from dark red (highest value) to dark blue (lowest value).

A second matrix was built considering only a 329-bp region of the *rpoB* gene (Fig. 2a and b) that was suggested by Ko et al. (31). The matrix returned an interspecies pairwise identity interval of 73.3% to 95.1%. The interspecies variation interval was between 4.9% and 26.7%. The intraspecies identity interval determined was 95.2 to 100%, resulting in an intraspecies variation interval between 0% and 4.8%. As previously described, these values permit the identification of isolates as belonging to the same species.

On the basis of the intra- and interspecies intervals calculated from the 329-bp *rpoB* gene region identity matrix, we analyzed the results for 14 isolates that showed discrepancies in *mip* and *rpoB* gene identification. The two L. anisa isolates determined according to the gold standard *mip* gene classification scheme were correctly identified; in contrast, the percentage of identity found for *rpoB* with respect to the reference strains (92.4%) did not fall within the intraspecies identity interval (lower cutoff at 95.1%), thus suggesting the possibility that the strains belong to different species. The same considerations can be applied to the four *L. quateirensis* isolates, which showed a percentage of identity for the *rpoB* gene of 94.5%.

The identity values of seven strains of *L. feeleii* and one isolate of *L. quinlivanii*, determined according to the *mip* gene classification scheme, showed borderline results with the *rpoB* classification scheme based on the observed cutoff values of 95.1% to 95.4% for the presumptive *L. feeleii* and 95.7% for *L. quinlivanii*. These findings provide further evidence of their misidentification and the necessity of further investigation.

Moreover, Table 2 reports the nucleotide and amino acid differences in the wild strains with respect to the corresponding reference strains. Interestingly, it is possible to note that all the wild strains presented nucleotide differences in both genes. Despite the *rpoB* gene being characterized as having greater genetic variability (number of DNA mismatches), the deduced amino acid sequences of the *mip* gene showed a higher number of amino acid substitutions. It is important to emphasize that all 14 isolates focused on in our study showed few amino acid substitutions in the *mip* gene, from 1 to 3; in contrast, regarding the *rpo*B gene, only five amino acid substitutions were reported in *L. taurinensis*.

**TABLE 2:** *Legionella* species found during environmental surveillance with the number of nucleotide (DNA) and amino acid (AA) differences from the type strain in the *mip* and *rpoB* genes

					No. of DNA and AA mismatches			
*Legionella* species	GenBank accession no.	Culture collection and type strain	*Legionella* isolate ID	*mip* and *rpoB* sequence identification	*mip*		*rpoB*	
					DNA	AA	DNA	AA
L. anisa	LNXS01000032.1	ATCC 35292 and WA-316-C3	MR 1–5, MR 7–9, MR 16, MR 18, MR 21, MR 31–33, MR 39–53, MR 65, MR 86–92, MR 98–99, MR 111–112, MR 115–121	L. anisa	0	0	0	0
			MR 6	L. anisa	1	0	2	0
			MR 54, MR 97	L. anisa	20	1	25	0
L. feeleii	NZ_LBHK01000054.1	ATCC 35072 and ATCC 35072	MR 69–73, MR 104	L. feeleii	11	1	15	0
			MR 123	L. feeleii	11	1	16	0
L. londiniensis	LNYK01000008.1	ATCC 49505 and ATCC 49505	MR 57, MR 95–96, MR 100–103	L. londiniensis	0	0	0	0
L. nautarum	LNYO01000023.1	ATCC 49506 and ATCC 49506	MR 11, MR 84, MR 105–110, MR 126–131, MR 133	L. nautarum	0	0	0	0
L. quateirensis	LNYR01000011.1	ATCC 49507 and ATCC 49507	MR 66–68, MR 85	L. quateirensis	11	2	18	0
L. quinlivanii	LNYS01000014.1	ATCC 43830 and CDC 1442-AUS-E	MR 36	L. quinlivanii	23	3	14	0
L. rubrilucens	LNYT01000018.1	ATCC 35304 and WA-270A-C2	MR 15, MR 17, MR 19–20, MR 22–25, MR 35–35, MR 61–64, MR 74–83, MR 122, MR 124	L. rubrilucens	0	0	0	0
L. steelei	LNYY01000006.1	ATCC BAA2169 and IMVS3376	MR 10	L. steelei	1	1	0	0
L. taurinensis	UGOZ01000001.1	NCTC 13314 and NCTC13314	MR 12, MR 14, MR 26–30, MR 37–38, MR 55–56, MR 58–60, MR 93–94, MR 113–114, MR 125, MR 132, MR 134	L. taurinensis	0	0	0	0
			MR 13	L. taurinensis	0	0	9	5
L. pneumophila	NC_002942.5	ATCC 33152 and Philadelphia 1	MR 135	L. pneumophila	6	0	4	0

**FIG 3:**
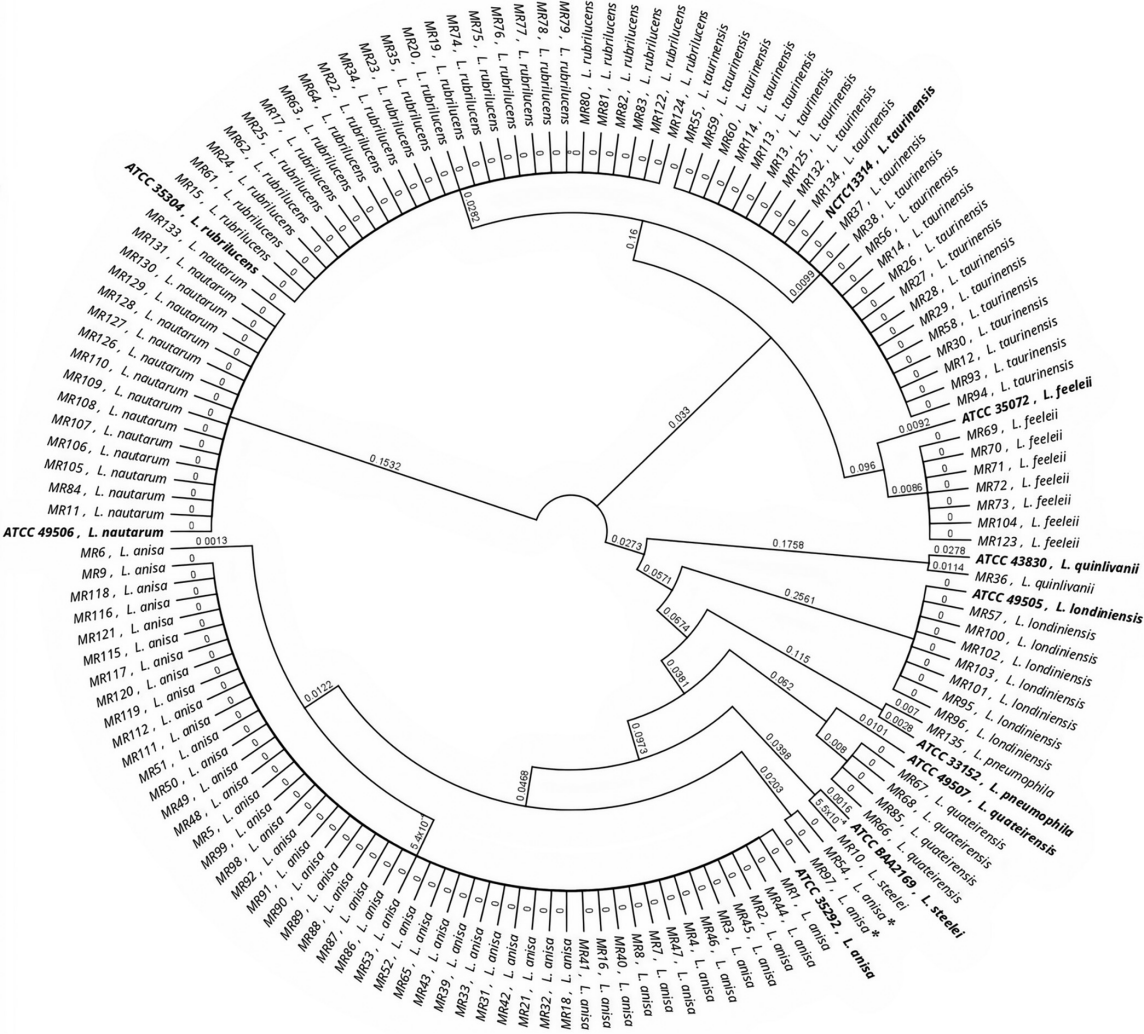
Analysis of the relationship among all 135 isolates and the respective type strains for the *mip* gene. Asterisks highlight the L. anisa isolates that diverge from the main branch. Reference strains are in bold.

**FIG 4:**
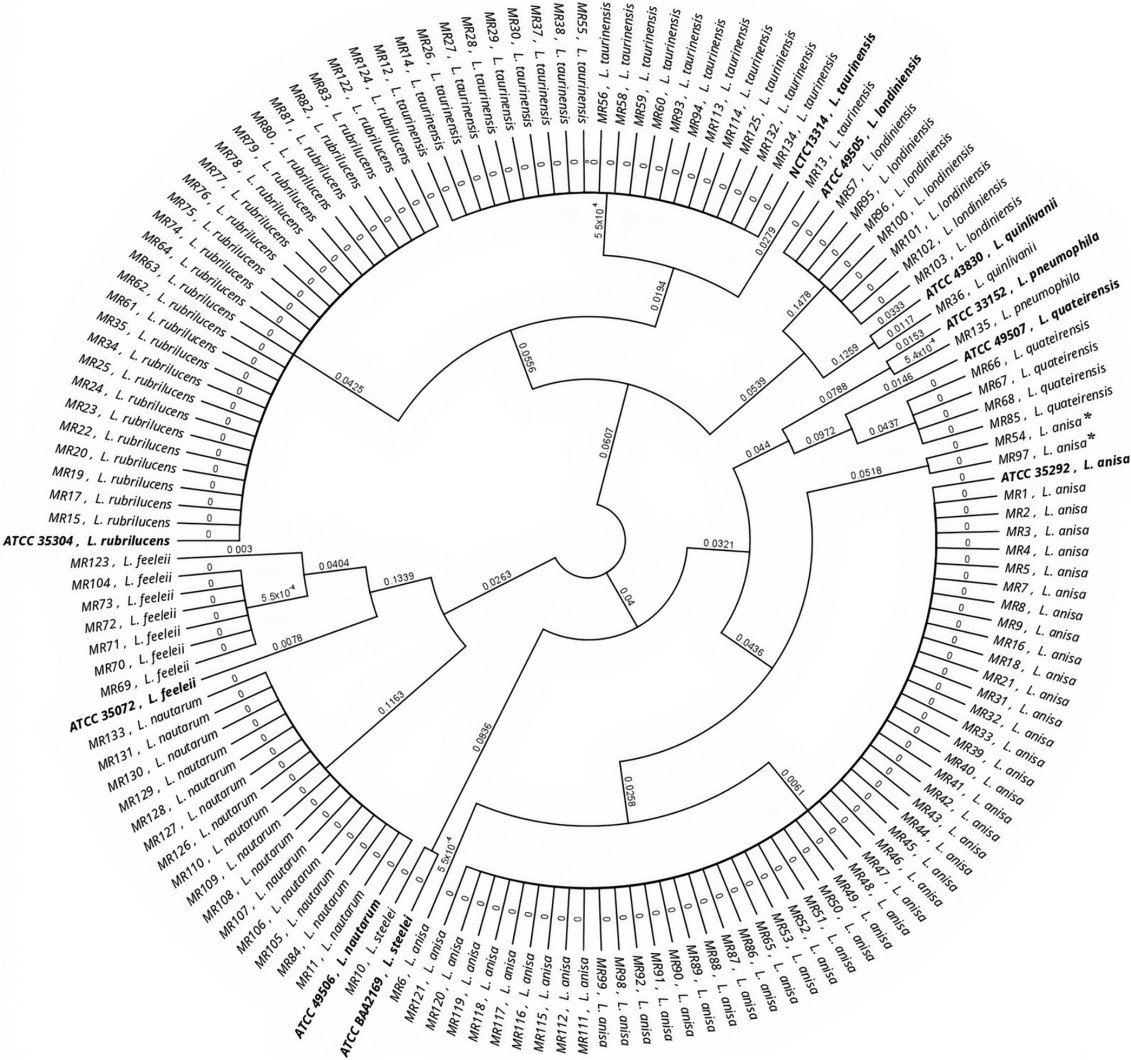
Analysis of the relationship among all 135 isolates and the respective type strain for the *rpoB* gene. Asterisks highlight the L. anisa isolates that diverge from the main branch. Reference strains are in bold.

Figures 3 and 4 display the relationship between all 135 isolates used in the study and the corresponding reference strains for the *mip* and *rpoB* genes, respectively. The dendrogram built using the *mip* and *rpoB* gene sequences regrouped all isolates into 10 clades corresponding to a specific *Legionella* species. In the *mip* gene dendrogram, no relevant differences were found, with the exception of two isolates of L. anisa (MR 54 and MR 97) that were separated from the corresponding main branch, suggesting a possible misidentification of these isolates. In contrast, the dendrogram built using the *rpoB* gene showed the same 10 clades but with a higher genetic distance between wild types and the reference strains.

**FIG 5:**
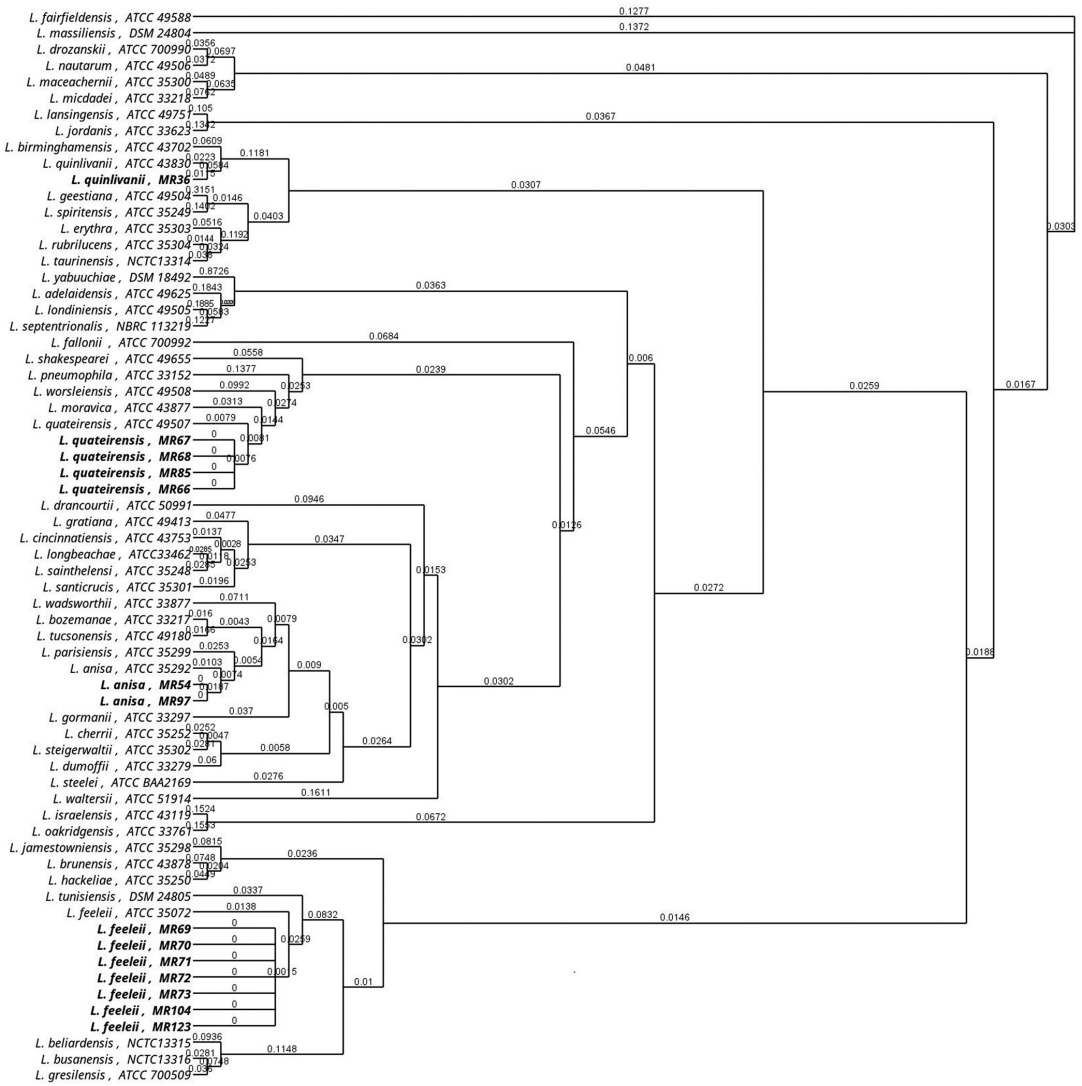
Phylogenetic dendrogram of *mip Legionella* sequences. Type strains versus wild strain isolates are in bold.

**FIG 6:**
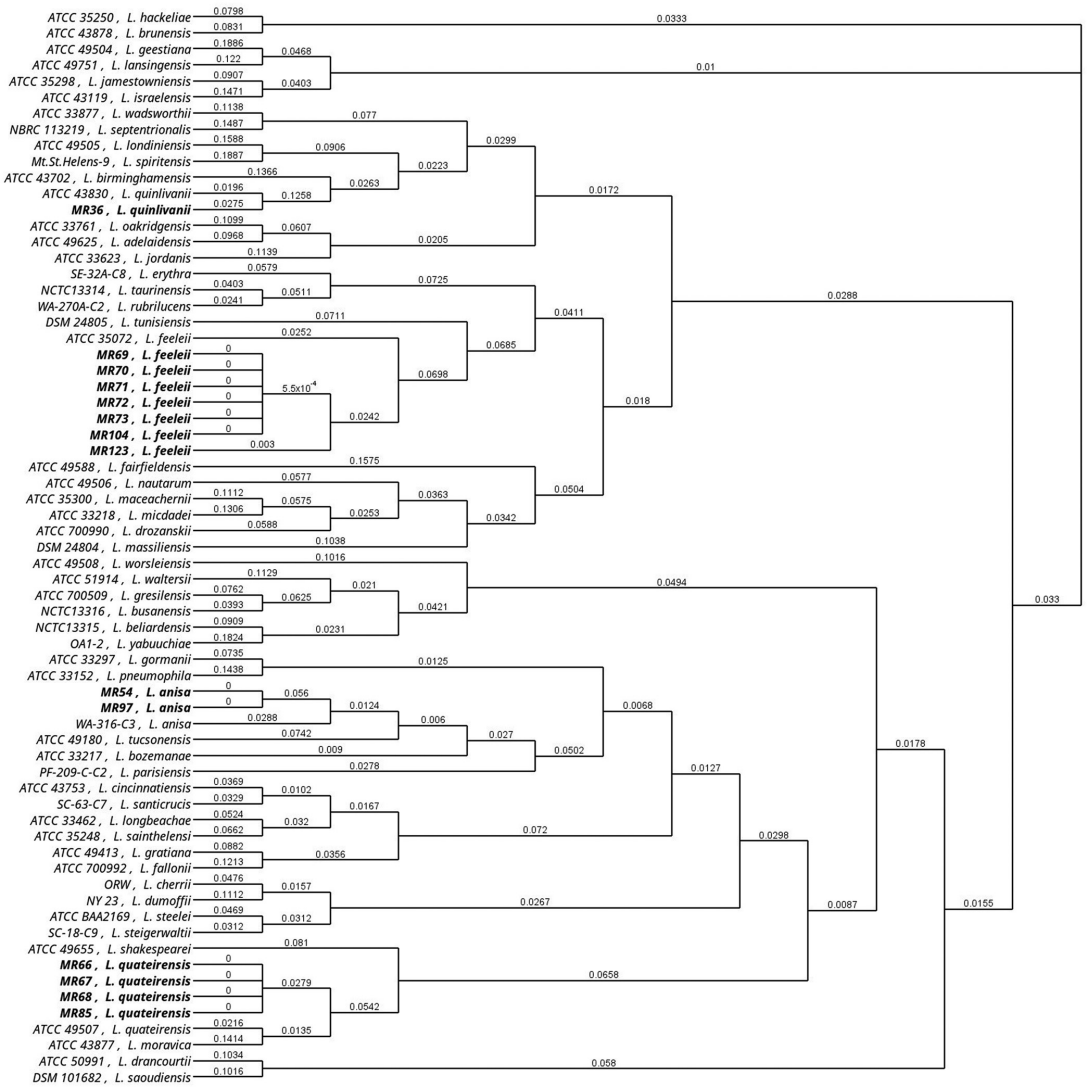
Phylogenetic dendrogram of *rpoB Legionella* sequences. Type strains versus wild strain isolates are in bold.

Figures 5 and 6 show detailed results for *mip* and *rpoB* genetic discrepancies between the 14 wild strains and their reference strains used in this study. The *rpoB* dendrogram showed that in the main clade of L. anisa, two isolates (MR 54 and MR 97) were different from others based on the *mip* gene dendrogram that was previously described. Moreover, in the *L. feeleii* clade, one isolate (MR 123) is separated from the main clade, and one isolate belonging to *L. quinlivanii* (MR 36) and four isolates belonging to *L. quateirensis* (MR 66, MR 67, MR 68, and MR 85) present differences from the corresponding reference strains ATCC 43830 and ATCC 49507, respectively.

## DISCUSSION

Several studies have compared molecular methods to detect *Legionella* spp. in environmental and clinical samples, and it is well known that the amplification and sequencing of some genes for the direct detection and identification of bacteria can be simple, convenient, and specific in their differentiation of bacterial species. The use of PCR methods in *Legionella* identification and typing, thanks to their species-specific capability, has increased the power to detect and identify species, reducing the time and cost compared to culture and antibody approaches as well as improving the sensitivity and specificity of identification, especially for clinical approaches.

Currently, non-*Lp* species have been mostly identified by only the *mip* gene, although several studies have shown that no single system is perfect and that other target genes need to be investigated (27). The use of a particular region of the *rpoB* gene was already tested to determine phylogenetic relationships as well as the identification scheme for enteric bacteria, Mycobacterium, *Bartonella*, and other microorganisms (30, 35, 36). Ko et al. have already shown that a partial region of *rpoB* is able to discriminate subspecies of *Lp* and several non-*Lp* species that have not been differentiated using the *mip* sequence classification scheme (37).

Many of the studies regarding the amplification of *rpoB* for *Legionella* spp. identification are exclusively focused on *Lp*, limiting the knowledge about the presence, distribution, and evolution of non-*Lp* species in the environment (37–39). This study showed the steps needed to build a new classification scheme using the *rpoB* gene and its application to a great number of non-*Lp* isolates (*n* = 135) distributed in both nosocomial and community environments. The results obtained were compared with the gold standard *mip* gene classification scheme already developed by Ratcliff et al. (26) and still in use by the European Society of Clinical Microbiology and Infectious Diseases (ESCMID) Study Group for *Legionella* Infections (ESGLI). Our results confirmed, in agreement with previous studies, that both *mip* and *rpoB* are able to discriminate among *Legionella* species, considering that our isolates (89.6%) showed complete concordance between the two classification schemes.

It is important to note that in some cases, there was no concordance between the *mip* and *rpoB* results, as there was a low percentage of genomic identity with respect to the reference strains for 14 isolates. In detail, our results suggest that sequencing using only *rpoB* is able to detect relevant genetic differences between the wild-type and the reference strains, which would otherwise be undetected using only the *mip* approach (e.g., *L. feeleii*, L. anisa, *L. quinlivanii*, and *L. quateirensis*). This result is especially interesting given that *L*. *quateirensis* and L. anisa showed a variability percentage for the *rpoB* gene outside the intraspecies interval of variation found here (0 to 4.8%); *L. feeleii* and *L. quinlivanii* had values very close to the variation cutoff, suggesting that the identification scheme using only one gene limits the discovery and study of species variation and sometimes limits discrimination between different species. In line with previous results, all the dendrogram representations show that there is lower genetic diversity in the *mip* gene between and within the clades; in contrast, the diversity in *rpoB* appears to be greater, leading to the identification of several isolates that showed evident differences from their respective clade or reference strain (e.g., L. anisa, *L. feeleii*, etc.). The results obtained using the *rpoB* gene seem to be useful for the identification of non-*Lp* species, and the results obtained permit the construction of the first *rpoB* gene classification scheme in the scientific literature.

Thanks to the matrices described above, we built pairwise identity intervals that allowed us to classify our unknown sequences based on comparisons with reference strains. The comparison carried out using the values obtained here seems reliable, and we propose that they be used in a classification scheme. For strains whose similarity percentages are very close to the cutoff values, further in-depth analyses are recommended. Based on the intervals of variation derived from the pairwise identity matrices, the discriminatory power of the 329-bp target region for the non-*Lp* species appears to be as reliable as that of the entire gene.

The comparison between the two matrices shows that the variability in the entire gene is greater than that in the selected region, suggesting that the analysis of a larger portion of the genome could increase the discriminatory power; therefore, approaches using new sequencing strategies, such as whole-genome sequencing (WGS), could contribute to better clarifying the identification of our isolates. This approach has already been applied to the four isolates of *L. quateirensis* described here. The average nucleotide identity (ANI) analysis, performed comparing their entire genome and the *L. quateirensis* type strain, showed pairwise values below the similarity threshold fixed to 95%, validating the hypothesis that the four strains belong to a presumptive new *Legionella* species (40).

In terms of the number of DNA and amino acid mismatches, most variability in the number of amino acid substitutions was observed in the *mip* gene, as all reported isolates showed discrepancies regarding the identification scheme based on the *mip* and *rpoB* genes. The role of the *mip* gene is widely documented; it is involved in the ability of L. pneumophila to replicate in eukaryotic cells and environmental amoebae (41). The substitutions found could interfere with pathways influenced by *mip*, as documented for *Lp* as well as for some non-*Lp* species (42–44).

It is possible to observe that the *rpoB* gene displayed a high number of DNA mismatches with a low number of amino acid variations. This result could be explained by the fact that *rpoB* is a housekeeping gene and that the alteration in the amino acid sequence could interfere with rifampicin resistance, as already demonstrated in other bacteria (e.g., Mycobacterium tuberculosis) and in a few *Legionella* species (39, 45). Therefore, the five amino acid mismatches found in *L. taurinensis* indicate the need to study the role of these variations in terms of protein function. Further investigations on *in silico* protein modeling and structural prediction other than biochemical functionality studies might contribute to better clarifying the role of these amino acid alterations and their evolution in *Legionella* species.

Although the non-*Lp* classification scheme using single-gene identification, such as the *mip* gene, is widely used and approved, the identification scheme for *Legionella* requires an update, such as introducing several patterns from various genes so as to increase the power of identification and improve phylogenetic studies. Especially for routine clinical and environmental laboratories where the whole-genome approach is expansive and laborious, the introduction of an easy, less expensive, and more sensitive scheme of identification could avoid errors in species characterization. Moreover, the proposed identification scheme could represent the first step toward acquiring information on different characteristics of isolates, such as changes in and development of antibiotics or disinfectant resistance, avoiding the failure of routine tests (e.g., urinary antigen test, serological and antibody-based assays), inadequate antibiotic treatments in human infection contest (e.g., rifampicin, fluoroquinolone, macrolides), and disinfection treatment. If a discrepancy is observed in this first step, then a more advanced technology, such as WGS, can be applied. This combined strategy represents an improved screening approach for *Legionella* isolate identification.

## MATERIALS AND METHODS

The isolates involved in this study come from *Legionella* environmental surveillance programs of several facilities commonly associated with the risk of *Legionella* infections, including hospitals, companies, and communities (e.g., hotels, private apartments).

### *Legionella* culture and isolate selection.

The *Legionella* culture technique was based on ISO 11731:2017 (20). The hot- and cold-water samples were sampled following the Italian National Unification and European Committee (UNI EN) ISO 19458:2006 (46) and Italian guidelines (19).

Different aliquots (from 0.2 to 0.1 mL) of the untreated, filtered, heated, and acid-treated samples were seeded on plates of the selective medium glycine-vancomycin-polymyxin B-cycloheximide (GVPC) (Thermo Fisher Scientific, Diagnostic, Ltd., Basingstoke, UK) and incubated at 35 ± 2°C with 2.5% CO_2_ for a maximum of 15 days. *Legionella* growth was evaluated every 2 or 3 days.

To confirm the presence of the *Legionella* genus, suspected colonies were subcultured on buffered charcoal yeast extract (BCYE) agar with (cys) and without (cys) l-cysteine (l-cys) supplementation (Thermo Fisher Scientific, Diagnostic, Ltd., Basingstoke, UK). Moreover, as a negative control, the same isolates were spread on tryptone soy agar (TSA) with 5% sheep blood agar (Thermo Fisher Scientific, Diagnostic, Ltd., Basingstoke, UK) and incubated under the same conditions previously described, as *Legionella* is not able to grow on this medium. Only the colonies that grew on BCYE cys^+^ agar were considered for the next steps of the study.

### Serological and biochemical typing.

The predicted *Legionella* colonies were then identified using the *Legionella* latex agglutination test (*Legionella* latex test kit, Thermo Fisher Scientific, Diagnostic, Ltd., Basingstoke, UK), which is able to distinguish between *Lp* and non-*Lp*. In particular, among *Lp*, it is possible to identify serogroup 1 (Sg1) from Sg2 to Sg14, while among non-*Lp*, it is possible to recognize only some non-*Lp*, such as L. anisa, *L. bozemanii* 1 and 2, Legionella gormanii, *L. longbeachae* 1 and 2, *L. dumoffii*, and *L. jordanis*. A total of 134 strains of non-*Lp* and 1 strain of *Lp* that was previously typed by sequence-based typing (SBT) and included as a positive control were selected for the study.

### Identification of *Legionella* spp. by *mip* and *rpoB* gene sequencing.

The DNA of each strain was extracted using the InstaGene purification matrix (Bio-Rad, Hercules, CA), and DNA concentrations were determined using a Qubit fluorometer (Thermo Fisher Scientific, Paisley, UK). PCR analysis for all non*-Lp* isolates was performed to determine the gene sequences of *mip* and *rpoB* as described by Ratcliff et al. (26) and Ko et al. (31), respectively.

*mip* gene amplification was performed using degenerate primers and modified by M13 tailing to avoid noise in the DNA sequence (47). *mip* gene amplification was performed in a 50-μL reaction mixture containing DreamTaq Green PCR master mix 2× (Thermo Fisher Diagnostics, Basingstoke, UK) and 40 pmol of each primer; 100 ng of the DNA extracted from the presumptive colonies was added as the template. The *mip* amplicons were sequenced using tailed M13 forward and reverse primers (*mip*-595R-M13R caggaaacagctatgaccCATATGCAAGACCTGAGGGAAC and *mip*-74F-M13F tgtaaaacgacggccagtGCTGCAACCGATGCCAC) to obtain complete coverage of the region of interest (47). Amplification was performed in a thermocycler under the following conditions: predenaturation for 3 min at 96°C, then 35 cycles consisting of 1 min at 94°C for denaturation, 2 min at 58°C for annealing, and 2 min at 72°C for extension, followed by a final extension at 72°C for 5 min. The reaction mixtures were then held at 4°C.

*rpoB* gene amplification was performed as described by Ko et al. (31). Gene amplification was performed in a 50-μL reaction volume containing 100 ng of template DNA, 40 pmol of each primer (RL1 5′-GATGATATCGATCAYCTDGG-3′; RL2 5′-TTCVGGCGTTTCAATNGGAC-3′), 1 U of *Taq* polymerase, and a PCR mixture consisting of PCR buffer 10×, 1.5 mM MgCl_2_, and 250 μM deoxynucleoside triphosphates (dNTPs). The thermal cycles consisted of 35 cycles, and each cycle consisted of 30 s at 94°C for denaturation, 30 s at 55°C for annealing, and 30 s at 72°C for extension, followed by a final extension at 72°C for 10 min.

PCR products were visualized by electrophoresis on a 2% agarose gel and stained with ethidium bromide. Following purification, DNA was sequenced using BigDye chemistry and analyzed on an ABI PRISM 3100 genetic analyzer (Applied Biosystems, Foster City, CA). Raw sequencing data were assembled using CLC Main Workbench 7.6.4 software.

The *mip* sequences were compared to sequences deposited in the *Legionella mip* gene sequence database using a similarity analysis tool. EWGLI has established an accessible web database (http://bioinformatics.phe.org.uk/cgi-bin/Legionella/mip/mip_id.cgi) that contains sequence data from described species and allows for the identification of non-*Lp* species. Species-level identification was performed on the basis of a similarity score of 98 to 100% compared to the sequences in the database (27) and considering the intra- and interspecies intervals of variation previously described by Ratcliff et al. (26).

The *rpoB* sequences were compared to type strain sequences deposited in NCBI from several culture collections, including the American Type Culture Collection (ATCC), National Collection of Type Cultures, Central Public Health Laboratory (NCTC), NITE Biological Research Center, National Institute of Technology and Evaluation (NBRC), and Deutsche Sammlung von Mikroorganismen und Zellkulturen (DSM). According to Adékambi et al. and Ko et al., the cutoff used for *rpoB* gene sequence-based identification was fixed at a 94 to 95% similarity percentage using an *rpoB* gene fragment of 300 to 600 bp (31, 48).

### Elaboration of matrices for the *rpoB* gene: definition of the intra- and interspecies intervals of variation.

*Legionella* type strains (*n* = 53) retrieved from the NCBI, were used to determine the ranges of the intra- and interspecies intervals of variation for the *rpo*B gene, resulting in a pairwise identity matrix for the entire gene with a length from 4,101 to 4,143 bp (Fig. 1a and b) and for the 329-bp selected region (Fig. 2a and b), corresponding to the amplicon suggested by Ko et al. (31). The list of type strains used in the study is reported in Table 3.

**TABLE 3:** NCBI type strains and wild strains used to build the pairwise identity matrix for intra- and interspecies interval determination in this study

Legionella species^*a*^	GenBank accession no.	Culture collection	Type strain
*L. adelaidensis*	LNKA01000005.1	ATCC 49625	1762-AUS-E
**L. anisa**	LNXS01000032.1	ATCC 35292	WA-316-C3
*L. beliardensis*	UGNV01000001.1	NCTC 13315	NCTC13315
*L. birminghamensis*	LNXT01000052.1	ATCC 43702	CDC 1407-AL-14
*L. bozemanae*	NZ_LBAW01000041.1	ATCC 33217	ATCC 33217
*L. brunensis*	LNXV01000034.1	ATCC 43878	ATCC 43878
*L. busanensis*	UGOD01000001.1	NCTC 13316	NCTC13316
*L. cherrii*	LNXW01000014.1	ATCC 35252	ORW
*L. cincinnatiensis*	LNXX01000018.1	ATCC 43753	CDC 72-OH-14
*L. drancourtii*	NZ_JH413847.1	ATCC 50991	LLAP12
*L. drozanskii*	LNXY01000006.1	ATCC 700990	ATCC 700990
*L. dumoffii*	LNXZ01000001.1	ATCC 33279	NY 23
*L. erythra*	LNYA01000024.1	ATCC 35303	SE-32A-C8
*L. fairfieldensis*	NZ_JHYC01000039.1	ATCC 49588	ATCC 49588
*L. fallonii*	LN614827.1	ATCC 700992	LLAP-10
***L. feeleii***	NZ_LBHK01000054.1	ATCC 35072	ATCC 35072
*L. geestiana*	LNYC01000041.1	ATCC 49504	ATCC 49504
*L. gormanii*	NZ_LBAY01000056.1	ATCC 33297	ATCC 33297
*L. gratiana*	LNYE01000004.1	ATCC 49413	Lyon 8420412
*L. gresilensis*	NZ_CAAAHX010000028.1	ATCC 700509	Greoux 11D13
*L. hackeliae*	NZ_LN681225.1	ATCC 35250	ATCC 35250
*L. israelensis*	CP038273.1	ATCC 43119	Bercovier 4
*L. jamestowniensis*	LNYG01000003.1	ATCC 35298	JA-26-G1-E2
*L. jordanis*	LNYJ01000005.1	ATCC 33623	BL-540
*L. lansingensis*	LNYI01000026.1	ATCC 49751	ATCC 49751
***L. londiniensis***	LNYK01000008.1	ATCC 49505	ATCC 49505
*L. longbeachae*	CP020412.2	ATCC 33462	ATCC 33462
*L. maceachernii*	NZ_FUXJ01000030.1	ATCC 35300	ATCC 35300
*L. massiliensis*	NZ_CCVW01000002.1	DSM 24804	LegA
*L. micdadei*	NZ_LN614830.1	ATCC 33218	ATCC 33218
*L. moravica*	LNYN01000019.1	ATCC 43877	ATCC 43877
***L. nautarum***	LNYO01000023.1	ATCC 49506	ATCC 49506
*L. oakridgensis*	NZ_LCUA01000039.1	ATCC 33761	ATCC 33761
*L. parisiensis*	LNYQ01000005.1	ATCC 35299	PF-209-C-C2
L. pneumophila	NC_002942.5	ATCC 33152	Philadelphia 1
***L. quateirensis***	LNYR01000011.1	ATCC 49507	ATCC 49507
***L. quinlivanii***	LNYS01000014.1	ATCC 43830	CDC 1442-AUS-E
***L. rubrilucens***	LNYT01000018.1	ATCC 35304	WA-270A-C2
*L. sainthelensi*	NZ_JHXP01000047.1	ATCC 35248	ATCC 35248
*L. santicrucis*	LNYU01000018.1	ATCC 35301	SC-63-C7
*L. saoudiensis*	NZ_LN901320.1	DSM 101682	LS-1
*L. septentrionalis*	NZ_RZGS01000010.1	NBRC 113219	Km711
*L. shakespearei*	LNYW01000039.1	ATCC 49655	ATCC 49655
*L. spiritensis*	LNYX01000029.1	ATCC 35249	Mt. St. Helens-9
***L. steelei***	LNYY01000006.1	ATCC BAA2169	IMVS3376
*L. steigerwaltii*	LNYZ01000025.1	ATCC 35302	SC-18-C9
***L. taurinensis***	UGOZ01000001.1	NCTC 13314	NCTC13314
*L. tucsonensis*	LNZA01000005.1	ATCC 49180	ATCC 49180
*L. tunisiensis*	NZ_CALJ01000292.1	DSM 24805	LegM
*L. wadsworthii*	NZ_JNIA01000004.1	ATCC 33877	ATCC 33877
*L. waltersii*	LNZB01000016.1	ATCC 51914	ATCC 51914
*L. worsleiensis*	LNZC01000014.1	ATCC 49508	ATCC 49508
*L. yabuuchiae*	NZ_CAAAIW010000035.1	DSM 18492	OA1-2

a In bold are reported *Legionella* species found during environmental surveillance.

The matrices were built using the multiple sequence comparison by log-expectation (MUSCLE) program (49) in Geneious Prime 2021.1.1 (https://www.geneious.com), retaining the default settings. The developed matrices permitted us to obtain a minimum and a maximum value of variation to establish intra- and interspecies intervals of divergence for the identification of the environmental isolates used in the present study. In detail, our 135 isolates were considered wild strains, and an in-house numbering scheme was used to label them (MR 1 to MR 135) (Table 4).

**TABLE 4:** GenBank accession numbers and ID labels of the *Legionella* wild strains used in this study

*Legionella* isolate ID	*Legionella* isolates	GenBank accession no.	
		*mip*	*rpoB*
MR1	L. anisa	MW021138	MZ367042
MR2	L. anisa	MW052865	MZ367043
MR3	L. anisa	MW052867	MZ367044
MR4	L. anisa	MW052869	MZ367045
MR5	L. anisa	MW052981	MZ367046
MR6	L. anisa	MW052872	MZ367047
MR7	L. anisa	MW052874	MZ367048
MR8	L. anisa	MW052875	MZ367049
MR9	L. anisa	MW052995	MZ367050
MR10	*L. steelei*	MW052877	MZ367051
MR11	*L. nautarum*	MW052931	MZ367052
MR12	*L. taurinensis*	MW052925	MZ367053
MR13	*L. taurinensis*	MW052973	MZ367054
MR14	*L. taurinensis*	MW052882	MZ367055
MR15	*L. rubrilucens*	MW052886	MZ367056
MR16	L. anisa	MW052879	MZ367057
MR17	*L. rubrilucens*	MW052895	MZ367058
MR18	L. anisa	MW052881	MZ367059
MR19	*L. rubrilucens*	MW052929	MZ367060
MR20	*L. rubrilucens*	MW052927	MZ367061
MR21	L. anisa	MW052885	MZ367062
MR22	*L. rubrilucens*	MW052914	MZ367063
MR23	*L. rubrilucens*	MW052919	MZ367064
MR24	*L. rubrilucens*	MW052890	MZ367065
MR25	*L. rubrilucens*	MW052893	MZ367066
MR26	*L. taurinensis*	MW052897	MZ367067
MR27	*L. taurinensis*	MW052901	MZ367068
MR28	*L. taurinensis*	MW052905	MZ367069
MR29	*L. taurinensis*	MW052908	MZ367070
MR30	*L. taurinensis*	MW052912	MZ367071
MR31	L. anisa	MW052891	MZ367072
MR32	L. anisa	MW052883	MZ367073
MR33	L. anisa	MW052894	MZ367074
MR34	*L. rubrilucens*	MW052917	MZ367075
MR35	*L. rubrilucens*	MW052921	MZ367076
MR36	*L. quinlivanii*	MW052923	MZ367077
MR37	*L. taurinensis*	MW052870	MZ367078
MR38	*L. taurinensis*	MW052873	MZ367079
MR39	L. anisa	MW052898	MZ367080
MR40	L. anisa	MW052876	MZ367081
MR41	L. anisa	MW052880	MZ367082
MR42	L. anisa	MW052887	MZ367083
MR43	L. anisa	MW052902	MZ367084
MR44	L. anisa	MW052863	MZ367085
MR45	L. anisa	MW052866	MZ367086
MR46	L. anisa	MW052868	MZ367087
MR47	L. anisa	MW052871	MZ367088
MR48	L. anisa	MW052982	MZ367089
MR49	L. anisa	MW052983	MZ367090
MR50	L. anisa	MW052984	MZ367091
MR51	L. anisa	MW052985	MZ367092
MR52	L. anisa	MW052906	MZ367093
MR53	L. anisa	MW052909	MZ367094
MR54	L. anisa	MW052913	MZ367095
MR55	*L. taurinensis*	MW052888	MZ367096
MR56	*L. taurinensis*	MW052878	MZ367097
MR57	*L. londiniensis*	MW052907	MZ367098
MR58	*L. taurinensis*	MW052910	MZ367099
MR59	*L. taurinensis*	MW052915	MZ367100
MR60	*L. taurinensis*	MW052920	MZ367101
MR61	*L. rubrilucens*	MW052889	MZ367102
MR62	*L. rubrilucens*	MW052892	MZ367103
MR63	*L. rubrilucens*	MW052896	MZ367104
MR64	*L. rubrilucens*	MW052900	MZ367105
MR65	L. anisa	MW052904	MZ367106
MR66	*L. quateirensis*	MW052978	MZ367107
MR67	*L. quateirensis*	MW052911	MZ367108
MR68	*L. quateirensis*	MW052916	MZ367109
MR69	*L. feeleii*	MW052922	MZ367110
MR70	*L. feeleii*	MW052924	MZ367111
MR71	*L. feeleii*	MW052926	MZ367112
MR72	*L. feeleii*	MW052928	MZ367113
MR73	*L. feeleii*	MW052930	MZ367114
MR74	*L. rubrilucens*	MW052933	MZ367115
MR75	*L. rubrilucens*	MW052934	MZ367116
MR76	*L. rubrilucens*	MW052935	MZ367117
MR77	*L. rubrilucens*	MW052936	MZ367118
MR78	*L. rubrilucens*	MW052937	MZ367119
MR79	*L. rubrilucens*	MW052938	MZ367120
MR80	*L. rubrilucens*	MW052939	MZ367121
MR81	*L. rubrilucens*	MW052940	MZ367122
MR82	*L. rubrilucens*	MW052941	MZ367123
MR83	*L. rubrilucens*	MW052942	MZ367124
MR84	*L. nautarum*	MW052944	MZ367125
MR85	*L. quateirensis*	MW052945	MZ367126
MR86	L. anisa	MW052946	MZ367127
MR87	L. anisa	MW052947	MZ367128
MR88	L. anisa	MW052948	MZ367129
MR89	L. anisa	MW052949	MZ367130
MR90	L. anisa	MW052950	MZ367131
MR91	L. anisa	MW052951	MZ367132
MR92	L. anisa	MW052952	MZ367133
MR93	*L. taurinensis*	MW052954	MZ367134
MR94	*L. taurinensis*	MW052955	MZ367135
MR95	*L. londiniensis*	MW052976	MZ367136
MR96	*L. londiniensis*	MW052977	MZ367137
MR97	L. anisa	MW052957	MZ367138
MR98	L. anisa	MW052958	MZ367139
MR99	L. anisa	MW052959	MZ367140
MR100	*L. londiniensis*	MW052960	MZ367141
MR101	*L. londiniensis*	MW052975	MZ367142
MR102	*L. londiniensis*	MW052961	MZ367143
MR103	*L. londiniensis*	MW052962	MZ367144
MR104	*L. feeleii*	MW052963	MZ367145
MR105	*L. nautarum*	MW052964	MZ367146
MR106	*L. nautarum*	MW052965	MZ367147
MR107	*L. nautarum*	MW052966	MZ367148
MR108	*L. nautarum*	MW052967	MZ367149
MR109	*L. nautarum*	MW052968	MZ367150
MR110	*L. nautarum*	MW052969	MZ367151
MR111	L. anisa	MW052986	MZ367152
MR112	L. anisa	MW052987	MZ367153
MR113	*L. taurinensis*	MW052972	MZ367154
MR114	*L. taurinensis*	MW052971	MZ367155
MR115	L. anisa	MW052991	MZ367156
MR116	L. anisa	MW052993	MZ367157
MR117	L. anisa	MW052990	MZ367158
MR118	L. anisa	MW052994	MZ367159
MR119	L. anisa	MW052988	MZ367160
MR120	L. anisa	MW052989	MZ367161
MR121	L. anisa	MW052992	MZ367162
MR122	*L. rubrilucens*	MW052979	MZ367163
MR123	*L. feeleii*	MW052974	MZ367164
MR124	*L. rubrilucens*	MW052980	MZ367165
MR125	*L. taurinensis*	MW052996	MZ367166
MR126	*L. nautarum*	MW052997	MZ367167
MR127	*L. nautarum*	MW052998	MZ367168
MR128	*L. nautarum*	MW052999	MZ367169
MR129	*L. nautarum*	MW053000	MZ367170
MR130	*L. nautarum*	MW053001	MZ367171
MR131	*L. nautarum*	MW053002	MZ367172
MR132	*L. taurinensis*	MW053003	MZ367173
MR133	*L. nautarum*	MW053004	MZ367174
MR134	*L. taurinensis*	MW053005	MZ367175
MR135	L. pneumophila	MW053006	MZ367176

### Phylogenetic and allelic diversity analysis.

To estimate the relationship among the *Legionella* isolates involved in the study, a multiple sequence alignment (MSA) and a phylogenetic tree were performed on the *mip* and *rpoB* gene sequences. For each taxon identified as previously described, the reference *mip* and *rpoB* gene sequences of the corresponding type strains from several culture collections were retrieved and added to the analysis (Table 3). When required, manual editing was performed on the sequences, trimming them to the same length as the reference sequence. The nucleotide sequences were aligned by the MUSCLE program. The obtained MSA was passed to FastTree (50), a tool for inferring approximate maximum likelihood phylogenetic trees. FastTree uses Jukes-Cantor as a genetic distance model and the Shimodaira-Hasegawa test to estimate the reliability of each split in the tree (51). Branch lengths were transformed to be equal, as in a cladogram. Branch labels display the substitutions per site. Both MUSCLE and FastTree were performed in Geneious Prime 2021.1.1 (https://www.geneious.com), retaining the default settings.

### Data availability.

The GenBank accession numbers of sequences generated during this study are listed in Table 4.
